# The Evolution of Sex Is Favoured During Adaptation to New Environments

**DOI:** 10.1371/journal.pbio.1001317

**Published:** 2012-05-01

**Authors:** Lutz Becks, Aneil F. Agrawal

**Affiliations:** 1 Department of Ecology & Evolutionary Biology, University of Toronto, Toronto, Ontario, Canada; 2 Department of General Ecology, Zoological Institute, Center for Biological Sciences, University of Cologne, Köln, Germany; University of Edinburgh, United Kingdom

## Abstract

Both theory and experiments have demonstrated that sex can facilitate adaptation, potentially yielding a group-level advantage to sex. However, it is unclear whether this process can help solve the more difficult problem of the maintenance of sex within populations. Using experimental populations of the facultatively sexual rotifer *Brachionus calyciflorus*, we show that rates of sex evolve to higher levels during adaptation but then decline as fitness plateaus. To assess the fitness consequences of genetic mixing, we directly compare the fitnesses of sexually and asexually derived genotypes that naturally occur in our experimental populations. Sexually derived genotypes are more fit than asexually derived genotypes when adaptive pressures are strong, but this pattern reverses as the pace of adaptation slows, matching the pattern of evolutionary change in the rate of sex. These fitness assays test the net effect of sex but cannot be used to disentangle whether selection on sex arises because highly sexual lineages become associated with different allele combinations or with different allele frequencies than less sexual lineages (i.e., “short-” or “long-term” effects, respectively). We infer which of these mechanisms provides an advantage to sex by performing additional manipulations to obtain fitness distributions of sexual and asexual progeny arrays from unbiased parents (rather than from naturally occurring, and thereby evolutionarily biased, parents). We find evidence that sex breaks down adaptive gene combinations, resulting in lower average fitness of sexual progeny (i.e., a short-term disadvantage to sex). As predicted by theory, the advantage to sex arises because sexually derived progeny are more variable in fitness, allowing for faster adaptation. This “long-term advantage” builds over multiple generations, eventually resulting in higher fitness of sexual types.

## Abstract 2

In experiments with a facultatively sexual rotifer, populations adapting to novel environments evolve higher rates of sex because sexual mixing quickly assembles well-adapted genotypes.

## Author Summary

For well over a century, biologists have wondered why sex is such a common mode of reproduction, given the immediate 2-fold fitness cost entailed by the reduced number of offspring per parent. The most classic explanation is that sex is favoured because it helps to generate the variation necessary for adaptation. While theoretical models and indirect lines of evidence support this idea, there are no direct experimental data and it is far from obvious whether any such advantage could balance the considerable costs of sex. Using experimental populations of a facultatively sexual species of rotifer, we demonstrate that rates of sex evolutionarily increase as populations adapt to novel environments. We show that sex creates a diverse array of genotypes, including many that are quite unfit but also others that are very fit in the new environment. Though the average fitness of these sexually derived offspring is lower than that of asexuals, those well-adapted genotypes generated by sex contribute disproportionately to future generations, causing the genetic propensity for sex to ultimately increase.

## Introduction

The pervasiveness of sex, given its varied and potentially large costs, is highly perplexing [1]–[10]. Numerous hypotheses have been proposed and sophisticated theoretical analyses have helped to define the conditions under which particular hypotheses may apply [3],[11]–[14]. Despite the importance of this problem, rarely have the hypotheses been tested by examining how key factors affect the evolution of sex [15]–[18].

Over a century ago, Weismann [19],[20] argued that sex might be beneficial because it helps generate the variation necessary for adaptation. While intuitively appealing, the idea is not necessarily correct as sex will increase the variance in fitness only if there is a preponderance of “negative genetic associations” such that good alleles are often found in genomes with bad alleles. It was later realized that such negative associations may develop under certain forms of nonlinear selection (as occurs when approaching an adaptive optimum [21]–[25]) or, perhaps more importantly, due to an interaction between directional selection and drift, known as the Hill-Robertson effect [6],[26]. For these more sophisticated reasons, Weismann's original conjecture is thought to be valid and is considered by many as the leading explanation for the evolutionary function of sex [27].

Rigorous theory shows that sex can facilitate adaptation [21],[24]–[26],[28], but the conditions under which this will translate into a net selective advantage for sex itself are more limited [21],[24],[29]–[34], especially given the infamous costs of sex [1],[3]. Indeed, a number of studies have demonstrated that sexual populations adapt faster than asexual populations [7]–[10],[35],[36]. Such studies imply a population- or group-level advantage to sex, though none of these studies directly competed sexual and asexual populations against one another during adaptation. Consequently, it is impossible to know whether any benefit to sex with respect to adaptation would have been outweighed by its immediate costs. More importantly, group-level advantages to sex cannot be used as evidence for the maintenance of sex within populations, as emphasized by John Maynard Smith [1] and George Williams [37].

Better support for adaptation providing a “gene-level” advantage to sex comes from survey studies showing that recombination tends to increase as an incidental by-product of directional selection on other traits [38],[39]. However, the evolution of recombination is not the same as the evolution of sex. The intrinsic costs of sex and recombination differ, and even ignoring these costs, theory shows that selection on recombination is often not an accurate predictor of selection on sex because of segregation effects [29],^40^,^41^. More evidence for adaptation favouring genetic shuffling comes from a recent study in *C. elegans* showing adaptation favours outcrossing over self-fertilization [42]. Though a related phenomenon, this is not direct evidence for the role of adaptation in maintaining sex. The contrast between selfing and outcrossing is not the same as the contrast between asex and sex because different types of genetic associations are involved. Further, the intrinsic costs of sex (relative to asexuality) differ from the intrinsic costs of outcrossing (relative to selfing). Despite these important differences, the recombination and outcrossing studies offer indirect evidence that adaptation can select for sex. However, direct experimental evidence for adaptation favouring sex is lacking.

Beyond the crucial step of empirically demonstrating the requirements necessary to cause an evolutionary increase in sex, a more thorough understanding requires identifying the population genetic mechanisms that drive the evolution of sex. A general theoretical framework divides the total selection on sex into components arising from “short-term” and “long-term” effects [30] (see [41],[43] for further discussion of these terms). The “short-term” effect of sex refers to the immediate fitness consequences of rearranging gene combinations. Sex does not directly change allele frequencies, but it does re-distribute alleles (i.e., breaks down genetic disequilibria). Whenever alleles interact to affect fitness (i.e., if there is dominance or epistasis), altering gene combinations will change fitness. For this reason, the mean fitness of sexual-derived progeny can differ from that of asexually derived progeny coming from the same set of parental genotypes. The short-term effect of sex results from alleles that promote sex being associated with different gene combinations than the alleles that promote asexual reproduction [10]–[12].

Regardless of whether there are gene interactions or not, the redistribution of alleles through sex can result in the variance of sexually derived offspring being different (higher or lower) than that of asexually derived offspring. If the sexually derived subpopulation has more variance than the asexually derived subpopulation, then the former will better respond to subsequent selection. Though sex does not immediately affect allele frequencies, it alters the genetic variance, which allows subsequent selection to cause allele frequencies to diverge between more versus less sexual lineages. The “long-term” effect of sex refers to selection on sex that results from genes that promote sex becoming associated with a different frequency of fitness-affecting alleles [10]–[12]. It is worth noting that the label “long-term” effect is somewhat misleading as long-term effects can arise over a single complete generation involving both reproduction and selection. While long-term effects can build in strength over multiple generations, it is not necessary to have hundreds of generations for this form of selection to alter the evolution of sex.

There is a myriad of hypotheses for the evolutionary maintenance of sex, but they can all be interpreted as providing an advantage to sex through either short- or long-term effects [11],[12]. Despite the importance of these general mechanisms to our understanding of selection on sex and the potential to study these effects by examining the effect of sex on the mean and variance in fitness, no empirical study has clearly linked the evolution of sex to either of these mechanisms.

Here we examine the Weismann hypothesis by evaluating whether sex is favoured during adaptation to a novel environment. We do this by (i) examining whether sex increases in frequency during adaptation and (ii) measuring the difference in fitness between naturally occurring sexual and asexual genotypes at various points during the course of adaptation. Finally, we test whether the advantage to sex arises from a short- or long-term effect by examining the effects of sex on the mean and variance in fitness at several points over the course of adaptation. This allows us to test the prediction that sex is favoured through a long-term advantage [24],[27],[31],[32].

To test Weismann's hypothesis at the within-population level, we used replicated experimental populations of the haplodiploid monogonont rotifer *Brachionus calyciflorus*. These rotifers are facultatively sexual, reproducing amictically at low densities but changing to mictic (sexual) reproduction in response to a chemical stimulus indicative of high density [44]. When stimulated, the amictic mothers produce daughters that develop into mictic females. Unfertilized mictic females produce haploid eggs that develop into males, and if young mictic females mate, her haploid eggs are fertilized and develop into resting eggs. Amictic females hatch from resting eggs when stimulated by environmental cues. Previous work with rotifers from this source population reveals there is substantial genetic variation in the strength of the stimulus needed to induce sex, thus allowing for the evolution of rates of sex [17]. Amcitic eggs develop within 1 d and the time the females start producing their first offspring is less than 24 h after hatching. Fertilized mictic eggs (resting eggs) from this population hatch spontaneously at a high rate under typical lab conditions (between 1 and 5 d after they are produced; see Material and Methods and [45]). From these observations, we approximate the mean time to complete an asexual generation to be ∼1.5 d. The “sexual cycle” takes ∼6 d but involves two generations (∼1.5 for the production of mictic females and then ∼4.5 d for sexually derived offspring to hatch and mature). Given that the overall rate of sexual reproduction is low, the average generation time is expected to be closer to 1.5 d than 4.5 d.

All of our replicate populations descended from a common natural source. However, 10 replicates came from subpopulations more recently adapted in the lab to one environment (“Environment A”), whereas 10 other replicates came from subpopulations more recently adapted to another (“Environment B”). The two environments differ in their algal food source and NaCl concentration. For our main experiment, 10 replicates (5 from each environment) serve as control (non-adapting) populations and are maintained under the environmental conditions to which they had already adapted. The remaining 10 populations are transitioned to the alternative environment (5 replicates A→B; 5 replicates B→A); we refer to these as “adapting” populations. This reciprocal experimental design offers the opportunity to infer the role of adaptation *per se*, rather than a particular environment, in affecting the evolution of sex. Population sizes are relatively large throughout the experiment (*N*≅3,500–7,500).

## Results

**Figure 1:**
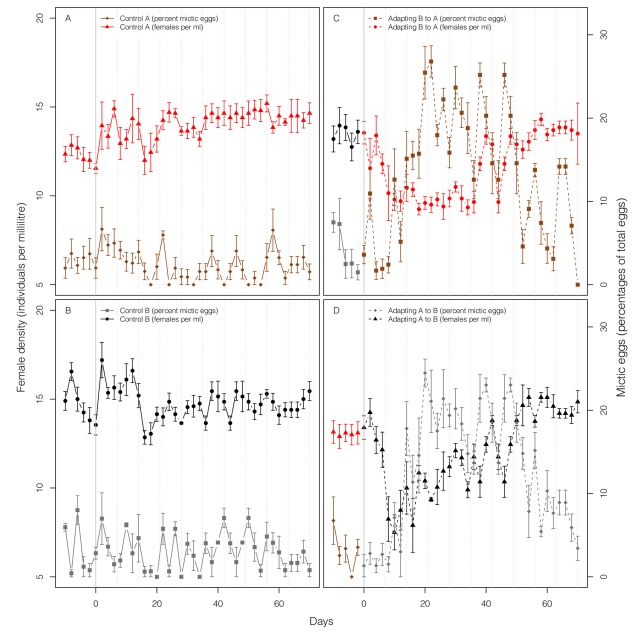
Female density and sexual investment in adapting and control populations. Replicated rotifer populations were kept for 80 d either in the environment to which they were previously adapted (non-adapting controls, A and B) or moved to a novel environment after 10 d (C and D). (A) Control populations for Environment A, (B) control populations for Environment B, (C) experimental populations adapting to Environment A (B→A); (D) experimental populations adapting to Environment B (A→B). Female density is shown as triangles for populations originating from Environment A (A and D) and as circles for populations originating from Environment B (B and C); points in red (black) represent measurements made in Environment A (B). The percentage of eggs produced by mixis is shown as diamonds for populations originating from Environment A (C and D) and as squares for populations originating from Environment B (B and C); points in brown (grey) represent measurements made in Environment A (B). Error bars denote ±1 standard error. In both environments, investment in sex (measured as percent fertilized mictic eggs) increases and then declines over time for adapting populations (quadratic term: B→A, χ^2^ = 106.66, *df* = 1, *p*<2.2×10^−16^; A→B, χ^2^ = 115.58, *df* = 1, *p*<2.2×10^−16^); there is no such pattern for control populations. Dotted vertical lines mark the time points at which the fertilized mictic and amictic eggs were sampled to compare the fitness of sexual and asexual genotypes (Figure 2) and to measure the propensity of sex (Figure 3).

**Figure 2:**
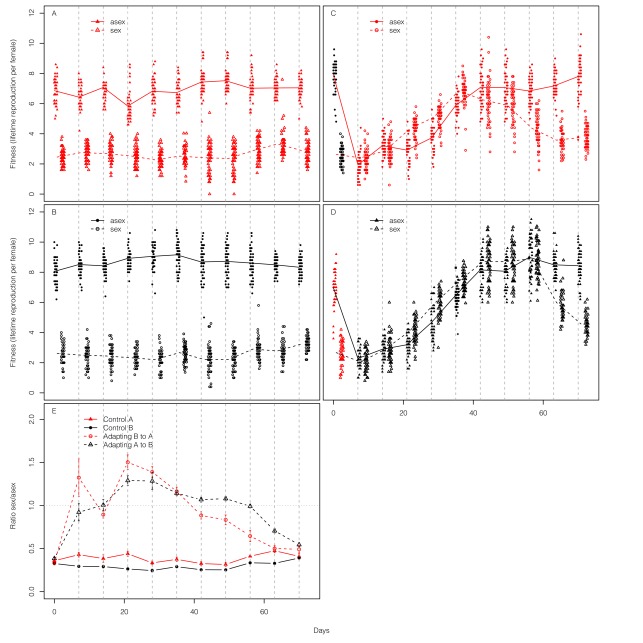
Fitness of sexually and asexually derived offspring from adapting and control populations. Naturally occurring sexual and asexual eggs are directly isolated from populations; lifetime reproduction is measured on the third clonal generation in the same environment from which the eggs are isolated (red, Environment A; black, Environment B). (A) Control populations in Environment A; (B) control populations in Environment B; (C) populations adapting to Environment A (B→A); (D) populations adapting to Environment B (A→B); sexual and asexuals were assayed at the same time as each other each week but points have been offset for clarity. Each data point represents the average number of offspring of five clonal individuals per genotype of third generation females that were hatched from eggs isolated from the experimental populations. In adapting populations, sexually derived offspring are significantly more fit than asexually derived offspring during the early stages of adaptation but become significantly less fit after adaptation plateaus (see Table S1 for statistics). (E) The ratio of the mean fitness of sexual and asexually produced offspring. Genotypes from sexual eggs are represented by open symbols (dashed lines connect mean fitness of five replicate populations across time points) and genotypes from asexual eggs are represented by filled symbols (solid lines connect mean fitness of five replicate populations across time points).

Over the course of 70 d (ca. 45 asexual generations) of evolution, there is clear evidence of adaptation in the populations that experience an environmental change. Population densities, which initially plummet during the transition to the alternate environment, increase to stable levels characteristic of well-adapted populations (Figure 1). Moreover, estimates of individual fitness show similar increases over time (Figure 2). In contrast, control (non-adapting) populations remain stable both in density and in fitness assay measures over this period.

As predicted by the Weismann hypothesis, rates of sex increase during the period of rapid adaptation. Later, sex declines as adaptation slows, presumably reflecting the intrinsic costs of sex outweighing the diminishing benefits of sex as the opportunity for adaptation declines. These temporal changes in sex are evident in two separate measures of sex. First, we use the fraction of fertilized mictic eggs (out of all eggs) as an in situ measure of sexual investment (fertilized mictic eggs are visibly distinct from other eggs). There is an obvious increase in the investment in sexual eggs during the period of rapid adaptation, followed by a decrease (see Figure 1 legend for statistics). This pattern cannot be explained by density effects directly triggering sex as the observed changes in sex go in the opposite direction from the well-known pattern for this species in which high density induces sex [44]. In contrast to the adapting populations, the percentage of fertilized mictic eggs in the control populations shows little change.

**Figure 3:**
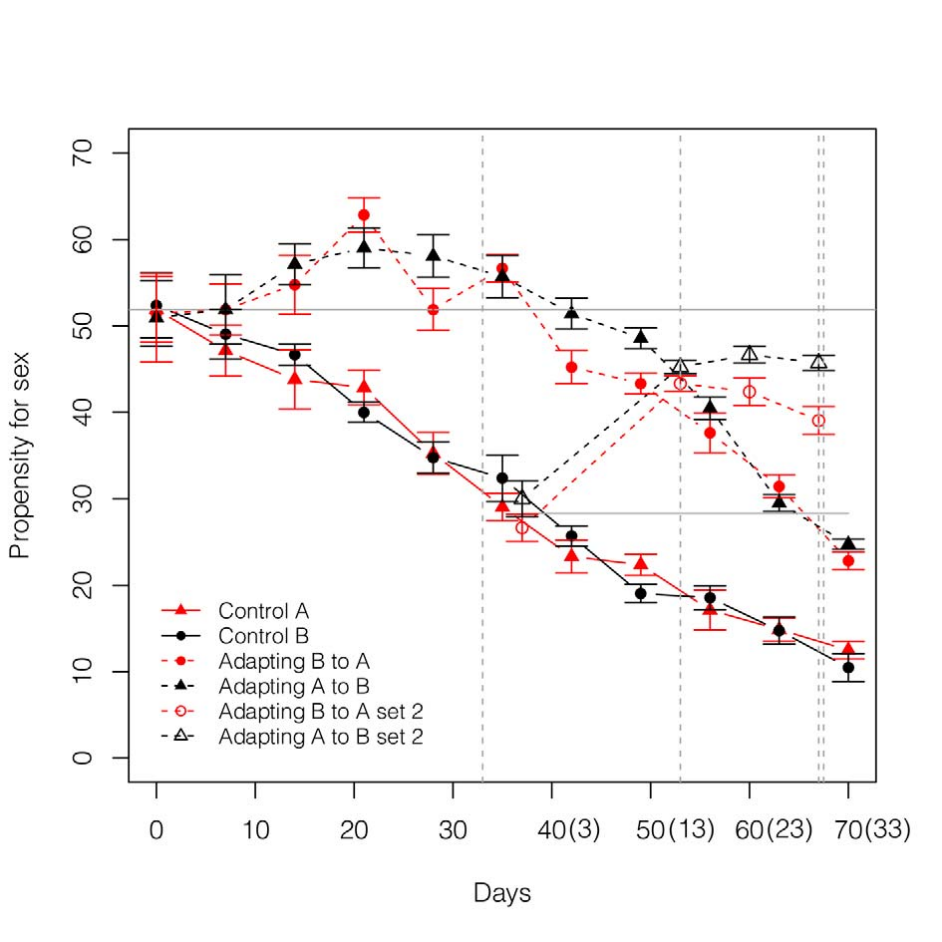
Evolution of the propensity for sex in adapting and control populations measured under standardized conditions. The propensity for sex is measured as percentage of females induced to sexual reproduction when exposed to a standardized stimulus. Data points represent the mean of five replicate populations per treatment ±1 standard error. The grey horizontal lines represent the initial propensity for sex and are shown for reference (upper line for Set 1; lower line for Set 2). The propensity for sex decreases in the control populations (solid lines) independent of the environment. In populations adapting to new environments (dashed lines), the propensity for sex increases and then declines (quadratic term: B→A χ^2^ = 18.5, *df* = 1, *p* = 1.7×10^−5^; A→B χ^2^ = 30.2, *df* = 1, *p*<2.2×10^−16^). On day 37, a second set of adapting populations were initiated from the controls. They also show an increase in sex (open symbols). The numbers in parentheses on the time axis denote the number of days since initiation of the second set. Note that colour always depicts the environment from which eggs were isolated and in which the assay was performed (red, Environment A; black, Environment B). Grey vertical lines denote time points when high- and low-density subpopulations were started to test for short- and long-term effects of sex (Figure 4).

Our second measure is based on a controlled assay of the propensity for sex. Each week, 42 rotifers are isolated from each population and maintained individually under standardized conditions for three clonal generations. Third generation individuals are exposed to a specified concentration of a sex-inducing stimulus. We determine the fraction of individuals that are induced into sexual reproduction by this cue. In the adapting populations, we observe a significant increase in the propensity for sex during the early phases of adaptation, followed by a subsequent decline (Figure 3, see legend for statistics). In contrast, the propensity for sex declines monotonically in the control populations. On day 37, a second set of 10 adapting populations (five for each environment) was initiated from the control populations. We refer to these as the “Set 2 adapting populations.” Our data on this second set are less detailed and over a shorter period, but these populations also show a similar increase in sex.

Several lines of evidence indicate that the changes in the propensity for sex (Figure 3) are not due to a plastic response stimulated by moving into a new environment. First, the assays are always performed in the third generation after isolation into standardized conditions so these changes cannot be due to the immediate shock of changing environments. Second, the Day 0 data represent assays on third generation clonal descendants of rotifers that have just transitioned to the alternative environment. As there is no difference in sex between control and adapting populations at this initial time point, it is clear that sex is not a stress-induced response resulting from a mismatch between genotype and environment. An unlikely third possibility is that the stress of a novel environment accumulates over multiple generations to induce the delayed rise in sex observed in Figure 3. As described in Figure S1, we tested this by transferring rotifers to the alternative environment and propagating them clonally for an extended period as individual lineages to prevent changes due to selection. Compared to rotifers maintained in the original environments, there was no change in the propensity for sex either in the short term or after a 16-generation delay (which corresponds to the same time period as the rise in sex observed in Figure 3).

The results of our in situ measure of “investment in sex” (Figure 1) and our well-controlled “propensity for sex” assay (Figure 3) are reasonably congruent for the adapting populations, but there is a puzzling inconsistency with respect to the controls. In the control populations, the “propensity for sex” declines monotonically, whereas the “investment in sex” is low and relatively constant. Because the strength of the sex-stimulating cue used in the “propensity for sex” assay is much stronger than the expected strength of the cue experienced in situ in the control populations based on their densities, we do not expect to see the same magnitude of change in the two types of measures. Nonetheless, some corresponding decline in the “investment in sex” measure is expected but not observed. As the data are somewhat noisy, it is conceivable that we simply lack the statistical power to detect a decline.

In this system, as in most others, the products of sexual reproduction are not phenotypically identical to those of asexual reproduction (i.e., fertilized mictic eggs are different than amictic eggs). Consequently, it is a concern whether changes in sex are actually due to selection for sex rather than a by-product of selection for some correlated feature. However, this alternative interpretation is inconsistent with our results. The parallel responses of adapting populations in both environments, as well as the pattern of temporal change within environments (increases during adaptation followed by decreases as fitness plateaus), indicate that neither environment favours resting (fertilized mictic) eggs per se.

Nonetheless, it would be more compelling to show differences in fitness between sexual and asexual genotypes to provide direct evidence of selection on the genetic consequences of sex. For this purpose, we sample fertilized mictic and amictic eggs weekly from each population. Rotifers are propagated individually before we measure lifetime reproduction for multiple clones of the third generation of each genotype, allowing us to compare recently created sexual and asexual genotypes that all develop from the same type of egg. The results, representing fitness measures on ∼22,000 individuals, are presented in Figure 2. In the control populations from both environments, genotypes derived from sexual reproduction are much less fit than those from asexual reproduction. In contrast, when populations transition to a new environment, we initially find no difference in fitness between sexually and asexually derived genotypes. As adaptation proceeds, sexually derived genotypes become significantly more fit than asexually derived genotypes (days 21–35 for A→B; days 21–49 for B→A). As populations approach their new fitness equilibrium, the pattern reverses again and the sexual load characteristic of well-adapted populations begins to re-emerge (days 42–70 for A→B; days 63–70 for B→A; see Table S1 for statistical comparisons between sexuals and asexuals at each time point).

The assays described above reflect differences in fitness between naturally occurring sexually and asexually produced offspring. The genotypes isolated for this assay are appropriately biased in that sexual genotypes will tend to descend from lineages with more sex in their history than asexual genotypes. This difference between the genealogical histories of naturally occurring fertilized mictic and amictic eggs is what yields a measure of the net effect of sex, but this also precludes a more detailed understanding of the population genetic mechanisms responsible. We cannot tell whether an observed advantage of sex results from the immediate benefit of genetic mixing (“short-term advantage”) or the accrued benefit of past selection on genetic variation released by previous bouts of sex (“long-term advantage”).

To differentiate between short- and long-term effects as mechanisms driving the evolution of sex, it is necessary to examine how sex affects the fitness of progeny from an unbiased set of parents. By comparing sexually and asexually derived offspring from random sets of parents, we can determine how sex affects the distribution of offspring fitness values without the confounding effects of past selection associated with sexually inclined lineages. By exposing a random sample of rotifers from each population to an extremely strong sex stimulus that induces sex at a very high rate across a wide array of genotypes [17], we obtain sexually derived offspring from a largely unbiased sample of parents. We obtain asexually derived offspring from random samples of rotifers from each population kept at low densities. Eggs are isolated and maintained individually under standardized conditions for multiple clonal generations before replicate measures of lifetime reproduction are made for each genotype. (This procedure is illustrated in Figure S2, where it is contrasted with the assay procedure for measuring fitness from naturally occurring sexual and asexual genotypes.) We perform this type of assay for the first set of adapting populations at two time points, sampling parents on day 33 (shortly after the propensity for sex has peaked) and day 67 (when adaptation is near complete and the propensity for sex is in decline). For the second set of adapting populations, we sample parents somewhat earlier during the course of adaptation (16 and 30 d after their initiation, corresponding to days 53 and 67 on Figures 2 and 3). We have analogous data for control populations for each of these time points.

**Figure 4:**
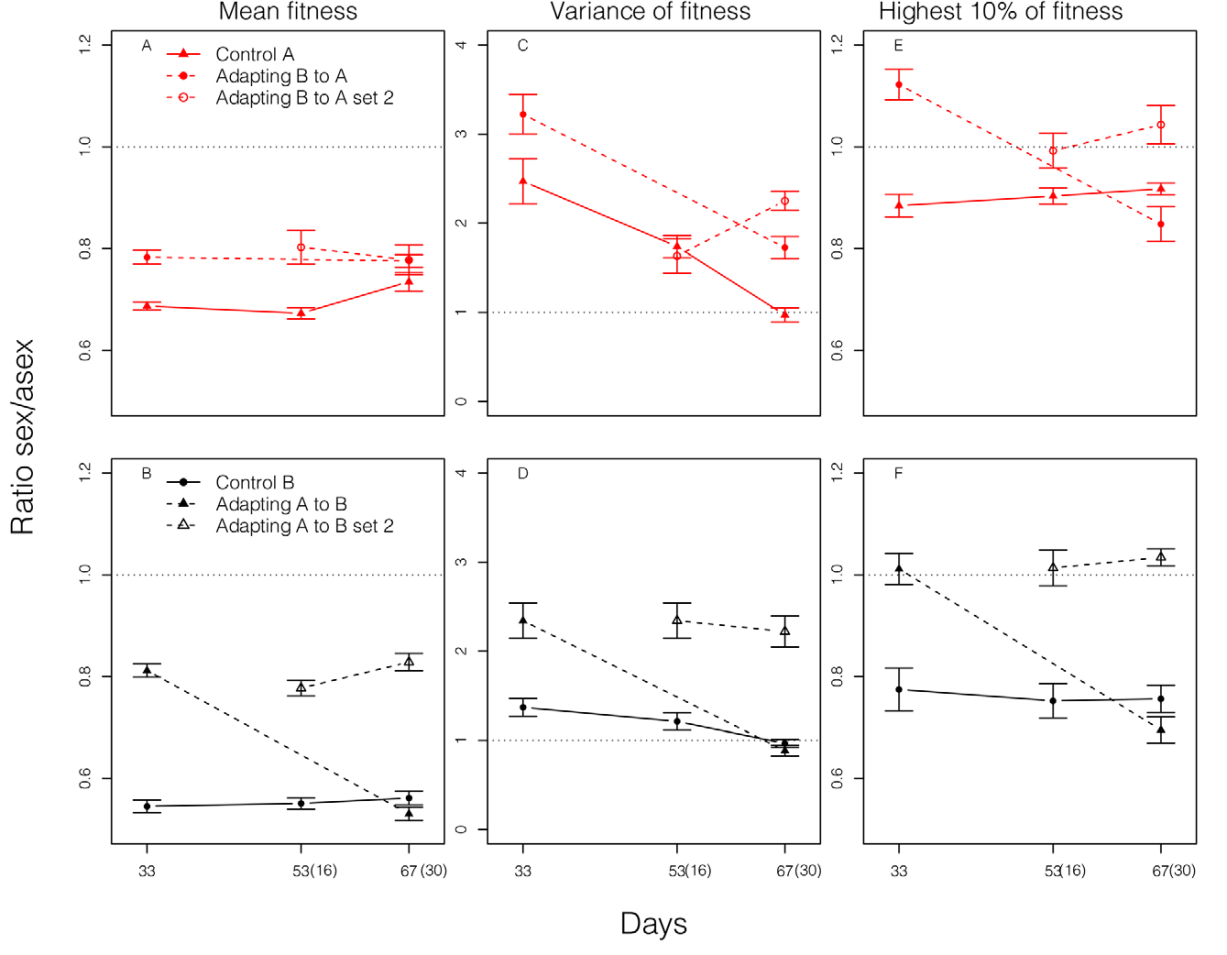
Short- and long-term effects of sex. Comparisons in the distributions of lifetime reproduction between sexually and asexually produced offspring from random sets of parents. Distributions are based on genotypic values, each measured as the mean of five clonal replicates. For the first set of adapting populations, distributions are measured at day 33 and 67; for the second set of adapting populations, distributions are measured at day 53 and 67, corresponding to 16 and 30 d after their initiation, as shown in parentheses. Control populations are measured at each time. (A, B) Ratio (± standard error) of average fitness of sexually produced offspring to that of asexually produced offspring. (C, D) Ratio of variance in fitness of sexually produced genotypes to that of asexually produced genotypes. Variance is calculated as the variance among genotypic means. (E, F) Ratio of the mean fitness of the top 10% of sexually produced genotypes to that of asexually produced genotypes.

The distributions of sexually and asexually derived offspring fitnesses are shown in Figures S3 and 4; ratios comparing key properties of these distributions are shown in Figure 4. Sexually produced offspring have lower mean fitness than asexually produced offspring (*t* = −18.9, *df* = 16, *p* = 2.3×10^−12^ for adapting populations; *t* = −62.8, *df* = 26, *p*<2×10^−16^ for control populations; Day 67 data from the first set of adapting populations are not used in these comparisons as fitness has plateaued before this point). The lower average fitness of sexually produced offspring is predicted whenever non-additive gene action (dominance and/or epstasis) plays an important role in shaping patterns of genetic associations (disequilibria) [3],[11]. Bad combinations of alleles that have been eliminated by past selection can be recreated by sex, reducing mean fitness, a phenomenon that can be thought of as a “sexual load” and is sometimes called “genetic slippage” [46],[47].

Although the distributions of sexually derived offspring fitness have lower averages than the corresponding distributions for asexuals, the variances for sexuals are higher (*t* = 17.0, df = 16, *P* = 1.1×10^−11^ for adapting populations; *t* = 6.1, df = 26, *P* = 1.8×10^−6^ for control populations). This increased variance associated with sex reflects the existence of negative genetic associations likely generated either by epistasis or Hill-Robertson effects [6],[26],[32]. Sex and recombination are expected to dissipate these disequilibria, resulting in an increase in genetic variance.

The pattern of sex reducing the mean but increasing the variance, indicative of a short-term disadvantage but a long-term advantage to sex, is qualitatively similar in both adapting and control populations. Although the directions of the short- and long-term effects are the same between treatments, the relative magnitudes differ. In Environment A, sex reduces mean fitness by ∼30% in control populations but only by ∼20% in adapting populations (before populations reach near complete adaptation). A similar effect occurs in Environment B, where sex reduces mean fitness by ∼45% in control populations but only by ∼20% in adapting populations. Thus, the short-term disadvantage of sex is ∼30%–50% smaller in adapting populations. This difference between adapting and control populations is supported by formal comparisons (*t* = −3.8, *df* = 16, *p* = 0.001 for Environment A; *t* = −14.8, *df* = 16, *p* = 9.6×10^−11^ for Environment B).

The long-term effect results from a difference in genotypic diversity in fitness created by sexual reproduction relative to that resulting from asexual reproduction. This is often discussed in terms of differences in variance. As described above, sexual genotypes are more variable in lifetime reproduction than asexuals in both adapting and control populations. The relative increase in variance due to sex is greater in adapting populations than in control populations (*t* = −2.4, *df* = 16, *p* = 0.03 for Environment A; *t* = −5.4, *df* = 16, *p* = 5.9×10^−5^ for Environment B; Figure 4C,D).

There are potential problems with using the variance as a measure of the long-term effect of sex when the mean fitness of sexually and asexually derived offspring differs. High variance of sexuals may result from the production of low fitness genotypes. The generation of such variants is not useful for adaptation and thus cannot contribute to a long-term advantage to sex. Rather, we are interested in whether sex tends to produce particularly good variants. For this purpose, we compare the average fitness of the top 10% of sexually and asexually produced genotypes (Figure 4E,F). Sex generates significantly better genotypes in the top end of its fitness distribution than does asexual reproduction in adapting populations (*t* = 3.06, *df* = 16, *p* = 0.007), but the opposite is true in control populations (*t* = −13.3, *df* = 26, *p* = 4.2×10^−13^). Similar patterns are observed using the top 5%, 15%, or 25% (see Figure S5).

Above, we have discussed fitness distributions for sexually and asexually derived offspring obtained in two different ways (Figure S2). First, we isolated naturally occurring fertilized mictic and amictic eggs, and thus, these two types of eggs came from lineages with different histories of sex (Figure 2). Second, we generated sexual and asexual offspring from random sets of parents (Figures 4, S3, S4, S5). If we compare these assays at a similar time point during adaptation (close to day 30), there is a dramatic difference. In the first assay (from non-random parents; Figure 2), we find that sexually derived offspring have higher average fitness than asexually derived offspring. In the second assay (random parents; Figure 4), sexually derived offspring have lower average fitness. As discussed above, even though sex produces offspring that are less fit, on average, than asexually derived offspring, sex also generates some particularly high fitness genotypes. These genotypes contribute disproportionately to future generations, carrying alleles for sex with them. As a result of generating extreme variants and subsequent selection, alleles that increase sexual propensity become associated with alleles conferring adaptation. Consequently, naturally occurring sexuals eventually become more fit, on average, than asexuals because of this accrued benefit from past selection. The higher average fitness of sexuals observed in the first assay midway through the course of adaptation represents the long-term advantage realized. However, as time passes, beneficial alleles will eventually accumulate in less sexually inclined genotypes, and thus the advantage to sex will erode as populations approach an adaptive optimum and the influx of new beneficial alleles slows. The short-term disadvantages of sex, along with other costs of sex, can then drive an evolutionary decrease in sex.

## Discussion

Previous experiments have shown that sexual groups can adapt faster, thereby providing indirect evidence of a group-level advantage to sex (at least in the absence of intrinsic costs) [7]–[10],[35],[36]. For the first time, we demonstrate that the frequency of sex within a population rises over time during adaptation. These results are consistent with the idea that Weismann's hypothesis can provide an advantage to sex at the gene level that can be sufficiently strong to overwhelm the intrinsic costs of sex. Weismann's hypothesis and related theories [27] make a strong prediction that sex should be favoured during adaptation because of a long-term advantage, and we have found evidence supporting this mechanism. On the other hand, much of this body of theory [29],[31]–[34] does not make a clear prediction with respect to short-term effects; when Hill-Robertson effects are responsible for negative disequilibria, short-term effects could be positive, negative, or zero, but models invoking non-linear selection to generate negative disequilibria predict negative short-term effects [24]. In our study short-term effects appear to be substantial. The reduction in negative short-term effects that seems to accompany the transition to a new environment (possibly reflecting environment-specific epistasis) is somewhat unexpected and reduces the threshold for a long-term advantage to create a net benefit to sex.

Though our results provide direct support for the operation of the Weismann hypothesis, we have not shown quantitatively that the Weismann hypothesis alone can fully explain the observed evolution of sex. It is possible that other factors also contribute to these changes. Here we consider two alternatives, but our data do not provide strong support for either. In this system, as in most others, the products of sexual reproduction are not phenotypically identical to those of asexual reproduction (i.e., fertilized mictic eggs are different than amictic eggs). Consequently, some of the observed evolution of sex could be a by-product of selection for fertilized mictic eggs (rather for genetic mixing). However, this alternative interpretation is inconsistent with our results. The parallel responses of adapting populations in both environments, as well as the pattern of temporal change within environments (increases during adaptation followed by decreases as fitness plateaus), indicate that neither environment favours resting (fertilized mictic) eggs per se. Moreover, we have direct evidence that genotype, independent of egg type, is important during adaptation; naturally occurring, sexually derived genotypes are more fit than asexually derived genotypes even when both develop from the same egg type (Figure 2).

A second factor of possible importance to our results is differential selection between the sexes [48]–[51]. In this system, (sexual) males are haploid, potentially allowing for more efficient selection on recessive beneficial alleles than can occur in the absence of sex. Under this hypothesis, we would expect that when we experimentally force individuals through the sexual cycle, the resulting offspring should, on average, be more fit than with asexual reproduction because of the extra sieve of haploid male selection that occurs incidentally during the process of creating sexual offspring. In fact, we observe the opposite; sexually derived genotypes from random sets of parents are less fit on average than asexually derived genotypes (Figure 4). This should not be taken as evidence that haploid selection has no effect at all, but rather it suggests that haploid selection does not play a strong role.

The costs of sex are expected to be high in this system, and it is unclear whether the observed benefits can outweigh these costs. In this regard, it is worth considering three points. First, modifier alleles that increase the rate of sex by a small degree experience only a small fraction of the cost of sex [52]. Second, long-term benefits can be quite powerful, especially when the baseline rate of sex is quite low [30],[52], as it is in our system (5%–7%, Figure 1). A modifier allele that slightly increases the rate of sex only suffers the cost of sex in those generations where it induces sex but enjoys the benefit of having created a good genotype for many generations.

Third, the advantage gained by “high-sex” genotypes during adaptation is likely considerably larger than it appears. The observed advantage in fitness of naturally occurring, sexually derived genotypes over asexually derived genotypes during adaptation reaches 30%–50% (Figure 2), but this underestimates the difference in fitness between “high-sex” genotypes and “low-sex” genotypes. This is because the distinction between “high-sex” genotypes and “low-sex” genotypes with respect to degree of sex is quantitative; both types use both reproductive modes. Consequently, the naturally occurring fertilized mictic eggs will come from both “high-sex” and “low-sex” parental genotypes but be biased toward coming from the former. Conversely, the amictic eggs will come from both “high-sex” and “low-sex” parents but be biased toward the latter. Thus, the difference in fitness between genotypes isolated from fertilized mictic eggs versus those isolated from amictic eggs will clearly underestimate the true difference in fitness between “high-sex” and “low-sex” genotypes.

The two environments used here were used in a previous study of the evolution of sex. The main result of that study was that higher rates of sex were maintained when populations experienced spatial heterogeneity in selection [17]. However, that experiment also provided a hint of the Weismann effect as even the spatially homogenous (control) populations showed an initial increase in sex followed by a decline on a time scale similar to that observed here. Because both environments were novel compared to the source population of rotifers, it is likely that the initial increase was due to an advantage to sex during adaptation to those environments. Though adaptation itself was not measured in that study, those results are consistent with what we have reported there.

Despite its importance to theory, the effect of sex on the distribution of offspring fitness has been measured in only a handful of taxa [45],[53]–[57]. In several of those cases [54],[55],[57], sex has been observed to reduce the mean but increase the variance, suggesting that long-term advantages to sex may be reasonably common but in none of those previous cases were evolutionary changes in the rate of sex measured. As seen here, short-term disadvantages coupled with long-term advantages can occur in cases where sex increases (adapting populations) as well as in cases where sex continuously declines (controls). However, we found substantial differences in the magnitudes of these effects between adapting and control populations. Moreover, the direction of the “long-term effect,” rather than just the magnitude, differs between adapting and control treatments if one considers the top 10% rather than the variance (the use of the latter is based on a weak selection approximation [30]).

While our experiment is unique in being able to link a change in sex to short- and long-term effects, a number of details remain unknown. A long-term advantage is expected to exist when sex dissipates negative genetic associations. Are negative genetic associations built by non-linear selection [21],[24],[25] or Hill-Robertson effects [6],[26],[32]? Similarly, we do not know whether dominance or epistasis is responsible for the immediate consequences of sex (short-term effects). Such information will be important to help understand the relative importance of segregation and recombination in driving the evolution of sex.

For sex to have any effect genetically, there must be genetic variation within populations. Even in well-adapted populations, we see clear evidence of genetic variance; when sex is imposed on random samples of parents, there is a dramatic decline in fitness. What sort of variation is responsible for this effect? One simple explanation is that recessive deleterious alleles hitchhike to high frequency in a heterozygous state and can persist as long as populations reproduce asexually much of the time so that deleterious homozygotes are rarely produced. A second explanation is that multiple high-fitness co-adapted genotypes are maintained by some form of balancing selection such as frequency-dependent selection. When it occurs, sex and recombination breaks down these co-adapted genotypes, resulting in low fitness genotypes. Unlike the first explanation, this alternative can apply to both haploid and diploid systems and so has been invoked to account for sex-induced reductions in fitness in studies on haploid *Chlamydomonas* [8],[54],[55],[58].

Though our experiment is consistent with the main tenets of the Weismann hypothesis, it also demonstrates a well-known weakness of this idea. The advantage to sex observed here is brief on an evolutionary time scale. Perhaps if adaptive optima are continually shifting, selection for sex could be maintained indefinitely [24]. Do selective pressures in nature change sufficiently frequently to explain the observed levels of sex? This is an empirical issue requiring data from the field. Lab-based studies such as the one reported here are necessary to directly evaluate the potential of hypotheses and to test their underlying mechanisms. However, such studies alone cannot prove any hypothesis as the explanation for the ubiquity of sex in nature. Attempts to study the evolution of sex in the field [15],[18],[53],[59] will be needed to evaluate the importance of results from theory and lab experimentation.

## Materials and Methods

The rotifers for this study descended from a population collected from sediment taken from Lake Onondaga, New York, in spring 2009 [45]. The populations used here were started from lab stocks that have previously been adapted to two different food conditions, Environments A and B (which we have previously called “low” and “high” food conditions [17]). These environments differ with respect to the algal suspension used to maintain the rotifers. Algae (*Monoraphidium minutum*, SAG 278-3, Algae Collection University of Goettingen) were taken from long-term chemostats to ensure constant food conditions over the course of the experiment. Chemostats were either run with a low nitrogen concentration in the medium = 160 µM (Environment A) or a higher nitrogen concentration in the medium = 1,000 µM (Environment B). The inorganic medium (nitrate as limiting N-source) was modified after [60], with additional 0.5 g/l NaCl to Environment A. The algae suspension for replacement of medium was prepared by diluting algae to concentrations of 2×10^6^ cells/ml with the same inorganic medium used for the chemostats but lacking nitrogen.

Stocks were kept under either of the two food conditions for 11 mo prior to the start of the experiment (∼9.5 mo with low rates of migration between the two environments—heterogeneous populations in [17]—and no migration for the last 6 wk before the start of the experiment; during this period, populations consisted of approximately 8,000 to 10,000 individuals), and experimental populations were started from these pre-adapted populations (10 populations per environment). Populations were maintained as semi-continuous cultures by replacing 10% of each culture including rotifers and algae every second day with a respective algae solution. Rotifer, amictic, and resting egg densities were enumerated under a stereoscope each time food was replaced [17]. Experimental populations of *Brachionus calyciflorus* were kept at 25±1°C (12/12 D-L) in tissue culture flasks (Sarsted, 500 ml) and moved randomly three times per week on the three shelves of the incubator. For more detailed methods, see Text S1.

### Adaptation

Replicate experimental populations were either maintained in the same environment to which they had previously adapted (*non-adapting control* populations; *n* = 5 per environment) or were transitioned to the other environment 10 d after the start of the experiment—that is, either from Environment A to B, or from B to A (*adapting* populations; *n* = 5 per environment). The transition occurred by substituting the other algae source during the regular food replacement schedule (see above). About 95% of the algae was replaced after 1 wk. Ten additional adapting populations (*n* = 5 per environment) were started at day 37 of the experiment. To create these populations, the 10% extracted media of the control populations on day 36 were pooled with others from the same environment and the following day were distributed among five new populations for each adapting population. The remaining volume was filled with fresh medium and the respective algae solution.

### Sex Stimulus in Standardized Environment

Sexual reproduction in *Brachionus* species is density dependent and stimulated by a chemical signal that is produced by the rotifers [61]. The propensity for sex was measured weekly and followed the protocol in [17]. Briefly, we isolated 42 asexual individuals from each population and individuals were transferred to single wells with 10 ml of food containing medium, so that each rotifer received the same food from which they were isolated. Individual rotifers were maintained under these conditions for two generations and one neonate of the third generation after isolation was individually transferred to a single cell of a 96-well plate with conditioned medium [17] containing the same food source from which they were isolated. The initial female was removed after they produced the first offspring and the offspring was scored as amictic or mictic by the type of offspring they produced. Sexual females produced only males (haploid) because they were unmated in the assay.

### Fitness Assay

Ten fertilized mictic (resting) eggs and 10 amictic eggs were isolated weekly from each population and transferred to a single well of a 24-well plate for hatching (Figure S2). The two types of eggs can be distinguished by their morphology: amictic eggs are completely filled and have a pale gray colour, while resting eggs are only partially filled and have a much darker coloration. Rotifer females from amictic eggs hatched within 1 d after isolation, and females from resting eggs hatched within 1 to 5 d. To avoid differences that could occur because sexually derived genotypes develop from resting eggs whereas asexually derived genotypes develop from amicitc eggs, we maintained each genotype by clonal reproduction for two generations prior to fitness measurements (in the same food environment from which they were isolated). The first five offspring from the third generation (asexual) after isolation were used to measure lifetime reproduction (five individuals per genotype). Each individual was placed in an individual well, and each day, the number of offspring was recorded and the female was transferred to a new well with fresh medium and food until the female died. Lifetime reproduction was used as a measure for fitness.

Spontaneously occurring fertilized mictic eggs are expected to originate from a non-random subsample of the population. To examine the effects of sex on a more random sample of genotypes, we transferred 5% of the populations to a new flask, added additional food, and allowed the population to grow to high densities (Figure S2), inducing almost the entire population to switch to sexual reproduction (density >30 females per ml; all genotypes are expected to switch to sexual reproduction at this density; cf. [17], Figure S2). Another 5% were transferred to a flask containing a large volume of medium and food, and these subpopulations were kept at low densities to ensure only asexual reproduction (less than one female per ml). After 7 d, 20 resting eggs were isolated from the high-density subpopulations and transferred individually to single wells for hatching and fitness assays as described above. Similarly, 20 amictic eggs were transferred from the low-density subpopulations. This procedure was applied to samples collected on Days 33 and 67 for the first set of adapting populations and on Days 53 and 67 for the second set of adapting populations. For each of these time points (Days 33, 53, and 67), similar data were collected from the non-adapting control populations.

### Data Analysis

Multivariate statistical analyses were done in the R statistical environment [62]. Treatment (Control A, Control B, Adapting B→A, Adapting A→B) specific models (generalized mixed models GLMM using the lmer4 package [63]) were used to test for differences in the percentage of fertilized mictic eggs (Figure 1) and propensity to reproduce sexually (Figure 3) with time as a fixed effect and replicate population as a random effect (using binomial error structure). To test for the increase and decrease in sex in the adapting populations, quadratic and linear models were compared.

The effect of sex on the distribution of genotype fitnesses was examined as follows. All analyses were performed on genotypic mean values (from five clonal replicates per genotype). Mean fitness of sexually and asexually derived rotifers hatched from naturally occurring eggs isolated directly from the experimental populations (Figure 2) were compared using environment and time-point-specific generalized mixed models (GLMM) with reproduction mode (sexually or asexually) as fixed and replicate population nested in reproduction mode as random effect.

To examine the effects of sex on a more random sample of genotypes, the distributions of sexually and asexually derived offspring were compared with respect to mean, variance, and mean of the top 10%. In each case, the data were analyzed with a linear model on the difference between sexuals and asexuals, using population as the unit of replication. To evaluate the effect of sex within treatments, we examined the significance of the intercept in separate analyses for adapting and control populations (variables were coded such that the intercept reflects the average effect across environments and time). For adapting populations, only Day 33 data for Set 1 were used as fitness had plateaued before Day 67 (Figure 2). For Set 2, we used the average values from Days 53 and 67 for each population (these represent days 16 and 30 of adaptation for Set 2). We obtained qualitatively similar results, using a total evidence approach by combining *p* values [64] from individual paired *t* tests (sex versus asex) for each set in each environment. To directly compare the effects of sex between adapting and control populations, we analyzed the difference in log of fitness between sexuals and asexuals in a linear model including both adapting and control treatments. Variance was calculated as the variance among genotypic means.

## Supporting Information

Figure S1 Propensity for sex measured 3 and 16 generations after switch of environments.
(DOC)

Figure S2 Fitness assays for naturally occurring asexually and sexually derived offspring (left) and asexually and sexually derived offspring from random set of parents (right).
(DOC)

Figure S3 Distribution of sexually and asexually derived offspring from random sets of parents from populations in Environment A.
(DOC)

Figure S4 Distribution of sexually and asexually derived offspring from random sets of parents from populations in Environment B.
(DOC)

Figure S5 Ratio of upper-percentiles for sexually and asexually derived genotypes obtained from random sets of parents.
(DOC)

Table S1 Summary of statistics for Figure 2.
(DOC)

Text S1 Detailed methods.
(DOCX)

## Abbreviations

GLMM: generalized mixed models
