# Supplementary material for: The Evolution of Sex Is Favoured During Adaptation to New Environments
Source: PLoS Biol. 2012 May 1;10(5):e1001317. doi: 10.1371/journal.pbio.1001317 (PMC3341334; doi:10.1371/journal.pbio.1001317)
Supplement: Figure S1 — Propensity for sex measured 3 and 16 generations after switch of environments. (DOC) [file pbio.1001317.s001.doc]

**Figure S1: Propensity for sex measured three and sixteen generations after switch of environments.** To test for immediate and delayed plasticity in sex, we used populations that descended from the "Set 2" adapting populations described in the main text. These populations had been maintained in either Environment A or B for 12 weeks prior to the assay reported here (i.e., were they well-adapted to their environment). We isolated 400 individuals from a population adapted to Environment A and 400 individuals from a population adapted to Environment B. These individuals were maintained individually in single wells for 16 generations, with half of the rotifers in their original environmental conditions and half in the alternative environmental conditions (i.e., 200 rotifers in each of the following treatments: A → A, A → B, B → B, B → A). These individual lineages were propagated clonally (i.e., no opportunity for selection) for a time period corresponding to the increase in sex observed in Figure 3. The propensity for sex is measured as percentage of females induced to sexual reproduction when exposed to a standardized stimulus (cue equivalent to ~ 22.5 females/ml)**.** There is little change in sex between the early assay performed after 3 generations (dark bars) or after 16 generations (light bars) or between rotifers kept in the original or alternative environments (compare A → A with A → B and B → B with B → A).
