# Supplementary material for: The Evolution of Sex Is Favoured During Adaptation to New Environments
Source: PLoS Biol. 2012 May 1;10(5):e1001317. doi: 10.1371/journal.pbio.1001317 (PMC3341334; doi:10.1371/journal.pbio.1001317)
Supplement: Figure S2 — Fitness assays for naturally occurring asexually and sexually derived offspring (left) and asexually and sexually derived offspring from random set of parents (right). (DOC) [file pbio.1001317.s002.doc]

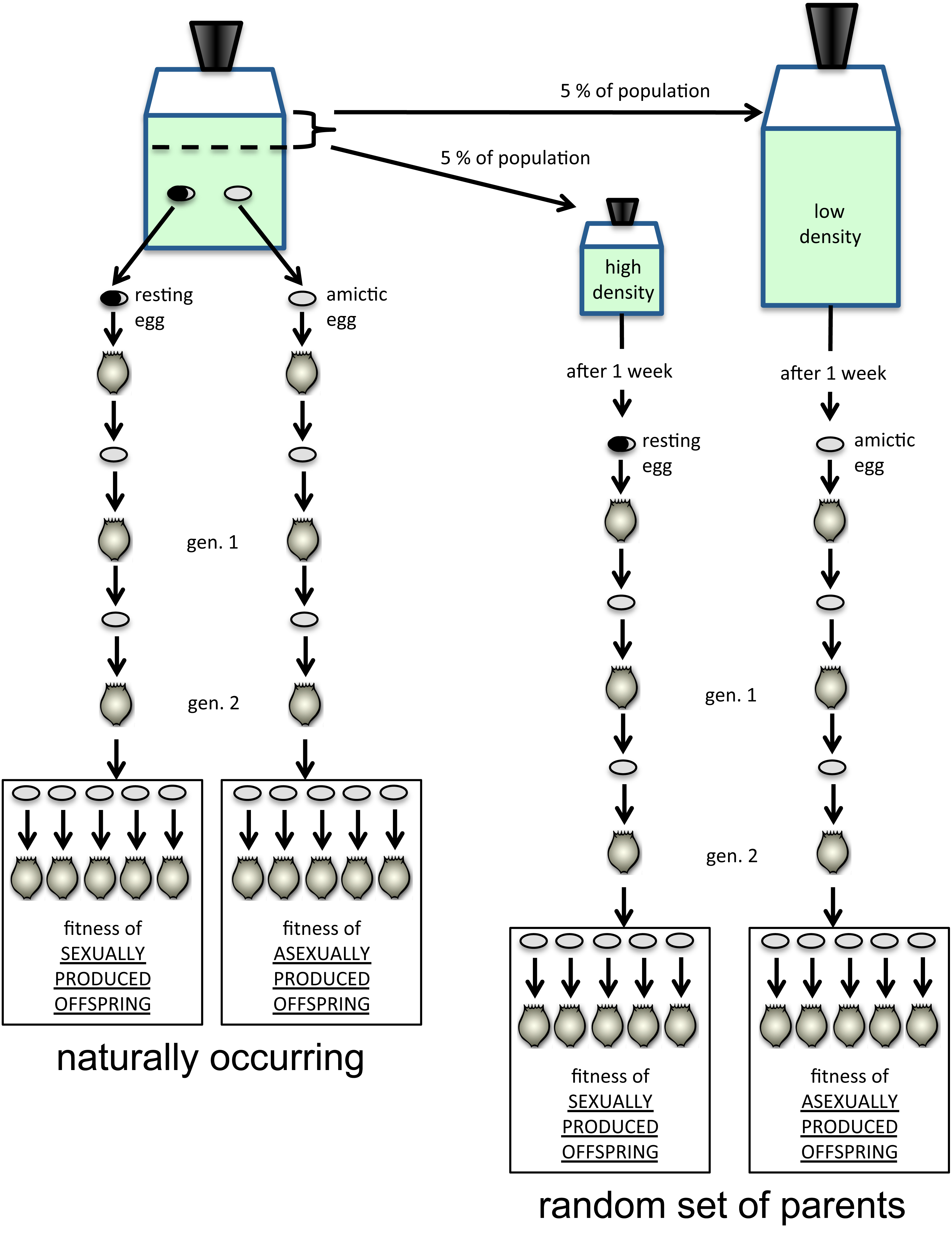


**Figure S2: Fitness assays for naturally occurring asexually and sexually derived offspring (left) and asexually and sexually derived offspring from random set of parents (right).** For the fitness assay for naturally occurring asexually and sexually derived offspring, eggs were isolated directly from the experimental populations (only one amictic and one fertilized mictic egg for one population shown here). The two eggs can be distinguished by their different morphology: amictic eggs are completely filled and have a pale gray color (asexually produced eggs) while fertilized mictic (resting) eggs are only partially filled and have a much darker coloration. Fitness measurements were preformed on five clonal individuals per genotype of the third generation after hatching from the eggs (Material and Methods). Fitness data from these assays are shown in Figure 2. To obtain sexually- and asexually-derived offspring from a random set of parents (see Results and Discussion), we transferred 5% from each experimental population to a new flask with a small volume (high-density populations) or large volume (low-density populations) with additional food. We allowed the populations in the small volume to grow to high densities for one week. Because the populations reached much higher densities in these populations than in the experimental populations, almost the entire subpopulations switched to sexual reproduction. We isolated fertilized mictic eggs (= sexually derived offspring from random set of parents) from these high-density populations and measured fitness in the same way as described above. For the low-density populations, we allowed the rotifers to reproduce for one week. Densities and thus sex-inducing stimuli were low and rotifers in these low-density populations reproduced exclusively asexually. We isolated amicitic eggs (= random asexually-derived offspring) from the low-densities subpopulations and measured fitness in the same fashion as described above. The data from this measurement are shown in Figures 4, S3-S5.
