# Supplementary material for: The Evolution of Sex Is Favoured During Adaptation to New Environments
Source: PLoS Biol. 2012 May 1;10(5):e1001317. doi: 10.1371/journal.pbio.1001317 (PMC3341334; doi:10.1371/journal.pbio.1001317)
Supplement: Figure S3 — Distribution of sexually and asexually derived offspring from random sets of parents from populations in Environment A. (DOC) [file pbio.1001317.s003.doc]

**Figure S3: Distribution of sexually- and asexually-derived offspring from random sets of parents from populations in Environment A.** Asexual (filled triangles) and sexual eggs (open triangles) were obtained from random samples of parents; lifetime reproduction was measured on the third clonal generation in Environment A: **A,B,C**) Control A; **D,E**) Adapting B → A; **F,G**) Adapting B → A (Set 2). Each data point represents the average number of offspring of five clonal individuals per genotype of third-generation females that were hatched from eggs isolated on Day 33 (left), Day 53 (middle) and Day 67 (right); for the second set of adapting populations at day 16 and 30 after their initiation (shown in parentheses). Solid lines connect the mean fitnesses of replicate populations for asexually-derived offspring and dashed lines connect the mean fitnesses of replicates for sexually-derived offspring. Replicate populations are shown in the same order for both sexual and asexual offspring.
