# Supplementary material for: The Evolution of Sex Is Favoured During Adaptation to New Environments
Source: PLoS Biol. 2012 May 1;10(5):e1001317. doi: 10.1371/journal.pbio.1001317 (PMC3341334; doi:10.1371/journal.pbio.1001317)
Supplement: Figure S4 — Distribution of sexually and asexually derived offspring from random sets of parents from populations in Environment B. (DOC) [file pbio.1001317.s004.doc]

**Figure S4: Distribution of sexually- and asexually-derived offspring from random sets of parents from populations in Environment B.** Asexual (filled triangles) and sexual eggs (open triangles) were obtained from random samples of parents; lifetime reproduction was measured on the third clonal generation in Environment B: **A,B,C**) Control B; **D,E**) Adapting A → B; **F,G**) Adapting A → B (Set 2). Other details as in Fig. S3.
