# Supplementary material for: The Evolution of Sex Is Favoured During Adaptation to New Environments
Source: PLoS Biol. 2012 May 1;10(5):e1001317. doi: 10.1371/journal.pbio.1001317 (PMC3341334; doi:10.1371/journal.pbio.1001317)
Supplement: Figure S5 — Ratio of upper-percentiles for sexually and asexually derived genotypes obtained from random sets of parents. (DOC) [file pbio.1001317.s005.doc]

**Figure S5: Ratio of upper-percentiles for sexually- and asexually-derived genotypes obtained from random sets of parents.**  Ratio (+/- standard error) of the top 5% (**A, B**), 15% (**C, D**), or 25% (**E, F**) of sexually-derived genotypes relative to that of asexually-derived genotypes obtained from random samples of parents (Figs. S3, S4). As in the case for the top 10% (Fig. 4E, F), sexual offspring are more fit than asexual offspring in adapting populations (P < 0.05) whereas sexuals are less fit in control populations (P < 0.001) for all three percentiles shown here.
