# Supplementary material for: The Evolution of Sex Is Favoured During Adaptation to New Environments
Source: PLoS Biol. 2012 May 1;10(5):e1001317. doi: 10.1371/journal.pbio.1001317 (PMC3341334; doi:10.1371/journal.pbio.1001317)
Supplement: Table S1 — Summary of statistics for Figure 2. (DOC) [file pbio.1001317.s006.doc]

**Table S1: Summary of statistics for Figure 2:** Fitness data were compared using environment and time point specific generalized mixed models (GLMM) with reproduction mode (sexually or asexually) as fixed and replicates nested in reproduction mode as random effect. See Figure 2 for the direction of difference in mean fitness. Asexuals are always more fit than sexuals in control populations but the difference in fitness changes sign for adapting populations. The degree of freedom is 1 for all comparisons.

|  | **Control A** |  |  |  |  | **Adapting B → A** |  |  |
| --- | --- | --- | --- | --- | --- | --- | --- | --- |
| **day** | **χ2** | **P value** |  |  | **day** | **χ2** | **P value** |  |
| 0 | 28.272 | < 0.005 | *** |  | 0 | 50.748 | < 0.005 | *** |
| 7 | 13.481 | < 0.005 | *** |  | 7 | 2.9605 | 0.08532 |  |
| 14 | 13.968 | < 0.005 | *** |  | 14 | 3.5791 | 0.05851 |  |
| 21 | 12.908 | < 0.005 | *** |  | 21 | 18.818 | < 0.005 | *** |
| 28 | 31.604 | < 0.005 | *** |  | 28 | 15.944 | < 0.005 | *** |
| 35 | 19.912 | < 0.005 | *** |  | 35 | 8.9292 | 0.002806 | *** |
| 42 | 27.694 | < 0.005 | *** |  | 42 | 11.782 | < 0.005 | *** |
| 49 | 29.395 | < 0.005 | *** |  | 49 | 11.615 | < 0.005 | *** |
| 56 | 17.921 | < 0.005 | *** |  | 56 | 16.362 | < 0.005 | *** |
| 63 | 23.685 | < 0.005 | *** |  | 63 | 24.407 | < 0.005 | *** |
| 70 | 19.109 | < 0.005 | *** |  | 70 | 24.055 | < 0.005 | *** |
|  |  |  |  |  |  |  |  |  |
|  | **Control B** |  |  |  |  | **Adapting A → B** |  |  |
| **day** | **χ2** | **P value** |  |  | **day** | **χ2** | **P value** |  |
| 0 | 15.381 | < 0.005 | *** |  | 0 | 34.133 | < 0.005 | *** |
| 7 | 18.283 | < 0.005 | *** |  | 7 | 2.3465 | 0.1256 |  |
| 14 | 19.867 | < 0.005 | *** |  | 14 | 0.067 | 0.7958 |  |
| 21 | 28.128 | < 0.005 | *** |  | 21 | 11.953 | < 0.005 | *** |
| 28 | 31.652 | < 0.005 | *** |  | 28 | 7.9651 | 0.004769 | *** |
| 35 | 29.245 | < 0.005 | *** |  | 35 | 16.535 | < 0.005 | *** |
| 42 | 26.635 | < 0.005 | *** |  | 42 | 5.9176 | 0.01499 | ** |
| 49 | 29.445 | < 0.005 | *** |  | 49 | 9.464 | 0.002095 | *** |
| 56 | 21.506 | < 0.005 | *** |  | 56 | 0.4326 | 0.5107 |  |
| 63 | 27.736 | < 0.005 | *** |  | 63 | 23.766 | < 0.005 | *** |
| 70 | 14.048 | < 0.005 | *** |  | 70 | 22.303 | < 0.005 | *** |
