# Supplementary material for: The Evolution of Sex Is Favoured During Adaptation to New Environments
Source: PLoS Biol. 2012 May 1;10(5):e1001317. doi: 10.1371/journal.pbio.1001317 (PMC3341334; doi:10.1371/journal.pbio.1001317)
Supplement: Text S1 — Detailed methods. (DOCX) [file pbio.1001317.s007.docx]

**Detailed methods**

A large amount of data was produced over the course of the experiment and many individual rotifers had to be maintained and propagated for several generations. We describe here in detail how this was done (LB performed the experiments). For the complete method section see main text.

Basic transferring of individual rotifers

Several of the assays involve counting and/or transferring of rotifers cultured in 24-well plates. The transfer of individual rotifers for the fitness assays and other assays could be done quickly because the number of rotifers per well is 0 (test clone dead), 1 (the test clone) or 2-4 rotifers per well (the test clone and the offspring produced within one day). Only the test clone had to be transferred and the number of offspring was usually instantly clear due to the low numbers of rotifers; no other information had to be recorded, (e.g., size, gender). All transfers were performed under a stereoscope using a pipette (Eppendorf) with a twice-bent pipette tip that allowed reaching into the well from above (rather than the usually used glass pipette with a rubber-ball that makes it often difficult to reach into a well). An additional advantage of using this method instead of a glass pipette is that a constant volume was transferred. Transferring of rotifers between plates typically took 60 to 90 seconds (24 individuals). Handling of rotifers in 96-wells for the propensity for sex assays took a bit longer.

Main fitness assays (on naturally-occurring sexual and asexual genotypes):

As described above, there are either 0 (individual test clone is dead), 1 (the individual test clone) or up to 4 rotifers (1 individual test clone and 3 offspring) in each well. The size difference between mother and offspring is large enough to identify the mother (we almost never encountered young females that carried eggs what would have made distinguishing between the mother and her offspring difficult; if we encountered two females carrying eggs in one well, the decision was based on the development stage of the egg she carries: the female with the more developed egg is the mother). Time to transfer rotifers from one 24-well to a new 24-well plate (60-90 seconds) + recording number of offspring produced and getting next 24-well plate (~60 seconds). In total, handling of 24 individual rotifers took ~2.0 to 2.5 minutes.

Each weekly fitness assay started with isolation of 200 asexual produced eggs (which hatched within 24h after isolation) and 200 resting eggs (hatched within 1 to 5 days after isolation; see below for isolation procedure). After hatching, the rotifers were kept individually and the first offspring produced by the hatched female was transferred and considered as generation one. Her first offspring was transferred representing the second generation after isolation. The initial five offspring that the second-generation female produced (in most cases one per day) were our test clones for the fitness assays. Five individuals per isolated genotype/clone were tested (5 * 200 clones from asexually produced eggs and 5 * 200 from sexually produced eggs). The number of rotifers that needed to be transferred decreased during the experiment due to death and the fact that older rotifers produce no more offspring. Rotifer lifespan varied between 1 and 14 days (the last days usually without producing offspring) and approximately 50% of the rotifers were dead at day 5 after the start of the fitness assay (and 95-100% dead around day 10). Typically, around 2000 rotifers were transferred per day (~. 3.75 h) with overlaps between fitness assays of two weeks, rarely three weekly assays.

Additional fitness assays (on sexual and asexual genotypes derived from “unbiased” sets of parents to infer short- and long-term effects):

We started two additional fitness assays at day 33, 53 and 67. For each of these, the eggs that we used were isolated from subpopulations that grew for one week before isolation of eggs. The actual work for the additional fitness assays started at day 40, 60 and 74 and followed the steps described above and in the main text. Again, the number of rotifers that needed to be transferred decreased during the experiment due to death and the fact that older rotifers produce no more offspring.

Propensity for sex

The propensity for sex assay involved four different steps that were performed on different days. In the first step we isolated the rotifers from the experimental populations. That was done either from the volume removed from experimental populations during the maintenance replacement or, if it was on a day without maintenance, from a small volume taken from the experimental populations (the same is true for the isolation of resting and asexually produced eggs from the populations). We isolate 840 rotifers each week which took ~ 2h. In the next steps, we removed the mother after she produced her first offspring and we repeated this till the second-generation produced the first offspring. The first offspring of the third generation was moved to the sex stimulus in 96-well plates and allowed to reproduce one daughter. We then remove the female that was exposed to the sex stimulus after she produced offspring (either sexual or asexual female). The last step was to check if males or females were produced, which was easily spotted by checking if there was one female with males (sex induced) or more than one female (no sex induced) in the well present. The number of males and the total number of females tested was recorded.

Maintenance of experimental populations:

Food suspension was prepared from the outflow of chemostats that ran stably over the course of the experiment. We counted the algae before preparing the food suspension using a haemocytometer. For each food type, we pooled the outflow of chemostats, filled the haemocytometer one time for each food environment (improved Neubauer, with a double chamber) and counted the number of algae and diluted the outflow accordingly with medium. Note that we could use the low number of replicated counts (two per food environment) because we used algae from chemostats that ran stable (i.e., only small fluctuations in the population densities over time).

Every other day, 10 % (50ml) of the 20 experimental populations were exchanged by an algal suspension. This was preformed in a laminar hood next to the incubators that we used for the experiment. We used twenty 50 ml centrifuge tubes placed in a rack to pour 50ml from each experimental population (50 ml marks on the tubes). We filled the missing volume of the experimental populations with food suspension to the 500ml mark of the tissue culture flask. Three 1 ml subsamples were taken from each 50ml falcon tube, transferred to wells of 24-well plates and counted. The whole procedure of population maintenance and determining population densities within the experimental populations took ~ 1.5h every other day. For the second set of adapting populations, no data on population densities were recorded and we only exchanged the medium.

Filling wells for fitness and propensity for sex assays:

We filled the well plates that were used for the propagation of the individual rotifers and the fitness and propensity for sex assays using either 10ml syringes to fill 1 ml into 24 well plates, using a multichannel pipette for the 96 well plates, or a beaker and marks on the 6 well plates at ~ 10ml. All wells of a plate were filled regardless if the test clone was already dead or not, so no time was used to check which wells had to be filled or not.
